# Synthesis of 14H-dibenzoxanthenes in green media using Sn(II)/nano silica as an efficient catalyst

**DOI:** 10.3389/fchem.2022.1015830

**Published:** 2022-11-02

**Authors:** Hossein Tavakol, Mahdieh Firouzi

**Affiliations:** Department of Chemistry, Isfahan University of Technology, Isfahan, Iran

**Keywords:** xanthene, silica, catalyst, green, naphthol

## Abstract

In this project, Sn(II)/nano silica has been prepared using a simple deposition of SnCl_2_.2.H_2_O on nano-silica. The prepared catalyst has been used as a green and reusable catalyst for synthesis of 14H-dibenzoxanthenes through a one-pot condensation reaction of β-naphthol with various aliphatic and aromatic aldehydes. Several xanthene derivatives have been synthesized using ethanol as the solvent, 10 mol percent of the catalyst, at reflux condition, in 3 h, and by 48%–94% yield. The structures of the synthesized derivatives are confirmed by melting point, FT-IR, ^13^C-NMR, and ^1^H-NMR analyses. Additionally, the nanocatalyst composition was confirmed by SEM, EDX, FT-IR, and XRD.

## Introduction

Xanthenes are one of the most important 6-membered, oxygen-containing heterocycles with high pharmaceutical activities (Silva et al., 2022). The synthesis of xanthene-based compounds was first reported by Bayer in 1987, when he synthesized fluorescein, a highly active fluorescent tracer, from resorcinol and phthalic anhydride (Shi et al., 1992; Shen et al., 2008). Xanthene derivatives have natural resources and are found in the soil and some plants such as *Indigofera Longeracemosa*, *Fabaceae*, and *Compositae* (Thangadurai et al., 2001). The xanthene derivatives are widely used in medicine, foods, and biological purposes. For example, erythrosine is a standard edible color in confectionaries (Jennings et al., 1990), and mangostin has anti-histamine, anti-inflammatory, and anti-cancer properties (POUPELIN et al., 1978; Shankaranarayan et al., 1979; Jung et al., 2006). Moreover, some uridine- and xanthene-based drugs are used for treatment of heart problems (Dai et al., 2022). They also have antioxidant (Liu et al., 2022) and antibacterial activities (Marona et al., 2009) and can be employed in biomembranes (Torrisi et al., 2022), dyes (Cesaratto et al., 2019), and solar cells (Maciejczyk et al., 2016). These structures have interesting deep-red and near-infrared adsorption potencies, which make them valuable candidates for cell imaging (Niu et al., 2016).

During the last century, various synthetic methodologies have been developed for xanthene-based structures. Subba et al. used mixed metal oxides as a catalyst to prepare dibenzoxanthene derivatives from β-naphthols and styrene oxides (Ganga et al., 2017). In a more complex structure, glutaraldehydes and aromatic β-ketosulfoxides were used for this purpose (Gabbutt et al., 1997). Monobenzoxanthenes have also been prepared from the reaction between 2-tetralone and various salicylaldehydes (Jha and Beal, 2004). Vazquez and Strongin reported phosphoric acid-catalyzed, 3-component reaction between resorcinol, 1,6-dihydroxynaphthalene, and benzaldehyde for preparation of xanthene derivatives (Sibrian-Vazquez and Strongin, 2009). Moreover, aminophenols, dimedone, cyclohexenone, and the β-naphthol–benzaldehyde couple have been used as other starting materials for synthesizing xanthenes (Lee et al., 2003; Ahadi et al., 2008; Strongin and Sibrian-Vazquez, 2009; Albadi et al., 2012). The crucial ingredient of these reactions is a catalyst. Therefore, various homogeneous and heterogeneous catalysts have been developed for these reactions, including CuO nanoparticles (Chaudhary et al., 2014), natural phosphates (Fallah et al., 2017), magnetite nanoparticles (Ghasemzadeh et al., 2013), ruthenium complexes (Tabatabaeian et al., 2016), functionalized imidazolium salts (Dutta et al., 2014), borane sulfonic acid (Dutta and Borah, 2015), and cellulose sulfuric acid (Azimi and Abbaspour-Gilandeh, 2016).

Among the various reported methodologies for the synthesis of xanthene derivatives, the reaction between β-naphthols and various aldehydes is the simplest way for this type of preparation. Taghavi and coworkers performed this reaction using orange peels as a heterogeneous catalyst (Taghavi et al., 2016). In another work, Zn/MCM-41 was used to catalyze this process for preparation of xanthene derivatives (Pirouzmand et al., 2016). Hosseini et al. used nano perlite sulfuric acid to synthesize different xanthene-based structures (Hosseini et al., 2016). In addition to the discussed methods, xanthene-containing structures have been synthesized from the aforementioned starting materials using copper aluminate nanoparticles, ferric salt-supported activated carbon, and montmorillonite in the reported studies (Mori et al., 2016; Huang et al., 2016, Z Fekri et al., 2016).

Here, to expand our experiences in the synthesis of valuable heterocycles and other organic structures (Keshavarzipour and Tavakol, 2016; Keshavarzipour and Tavakol, 2017; Shahabi and Tavakol, 2017) and to develop a new method for this reaction, an environmentally compatible, low-cost, non-toxic, and easily separable catalyst will be introduced to synthesize 14H-dibenzoxanthene derivatives from β-naphthol and aldehydes. This work reports a simple and efficient method for the preparation of dibenzoxanthene derivatives using a green solvent and an available catalyst. The used catalyst was not expensive and could be prepared easily. The large number of prepared derivatives shows the versatility of the employed method, in which different substituent and starting materials can be converted to the product *via* this method. In the following sections, the details of the preparation of Sn(II)-supported nano SiO_2_ and the use of it as a green and reusable catalyst for synthesis of 14H-dibenzoxanthenes through a one-pot condensation reaction will be presented in detail.

## Materials and methods

### Materials and instruments

All starting materials, solvents, and TLC plates (TLC-silica gel-f 454 60 nm) were bought from Merck Co. The compounds were of reagent grade (98% purity or higher) and used without further purifications. Melting points are related to the purified products. All the products were known compounds, and their structures were confirmed by comparing between their melting point and spectral data (IR, ^1^H-NMR, and ^13^C-NMR) with the reports. The melting points were recorded using Melt-temp apparatus with the end-capped capillary pyrex tube. FT-IR spectra were recorded in a JASCO instrument using a KBr pellet. ^1^H- and ^13^C-NMR spectra were recorded on a Bruker Ultrashield 400 instrument (400 MHz for 1H-NMR and 100 MHz for 13C-NMR) using CDCl_3_ as the solvent. The obtained FIDs were analyzed and interpreted using MestReNova software. The MIRA3TESCAN-XMU electronic microscope was used to obtain FESEM images and to perform EDS analyses. XRD patterns were obtained using a Philips X’PERT MPD instrument at 30 mA current and 40 kV electric potential. The XRD results were prepared with X’PERT High Score software.

### The preparation of Sn(II)/nano silica

0.28 g SnCl_2_^.^2H_2_O was added to a well-mixed suspension of 3 g nano-silica (40–50 nm) and 25 ml methylene chloride in a 50-ml beaker. The suspension was rigorously stirred at room temperature for 24 h. Then, the mixture was filtered and the filtrate was washed with 2 × 10 ml methylene chloride. The remaining solvent was removed using vacuum distillation, and the catalyst was placed in an electric oven at 100°C temperature for 2 h to obtain the final catalyst.

### The general procedure for preparation of 14H-dibenzoxanthenes

In a 25-ml single-necked flask, equipped with a condenser, 2 mmol of naphthol (or its derivative) and 1 mmol of aliphatic or aromatic aldehyde were added to the mixture of 10 ml ethanol and 0.1 mmol (10 mol%) of the catalyst (based on Sn). Heating was started, and the mixture was stirred for 3 h in the reflux condition. The progress of the reaction was followed by TLC using ethyl acetate: hexane (1:3) as the eluent. After completion of the reaction, the hot reaction mixture was filtered (hot-filtration) to remove the catalyst. Then, 20 ml of distilled water was added, and the raw product was extracted using 3 × 10 ml diethyl ether. The solvent was evaporated, and the product was purified using recrystallization in ethanol. The spectral data of the products are provided in Supporting Information.

## Results and discussions

**FIGURE 1:**
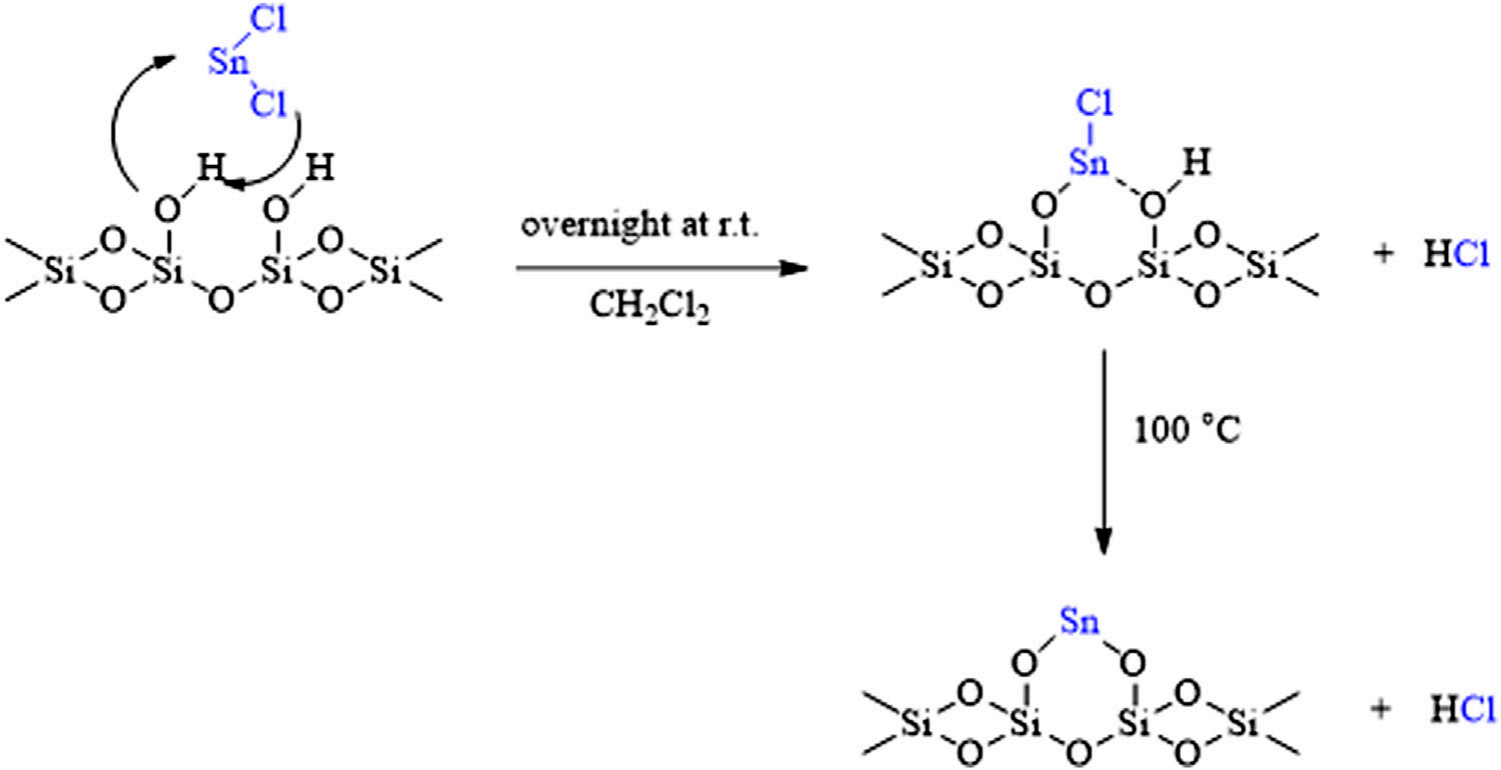
Chemical description of catalyst preparation.

The prepared catalyst (Sn(II)/nano silica) was produced by simple substitution of the surface hydroxy groups of silica instead of one or two chlorine atoms of SnCl_2_. As shown in Figure 1, the resulting catalyst consists of Sn(II) ions on its surface which can actively participate in the catalytic process. The placing of tin on the surface of nano-silica prevents aggregation of the catalyst particles and provides a higher availability for the catalyst. However, later, the presented EDS values showed the presence of chlorine atoms. Therefore, the whole process shown in Figure 1 is not completed in all sites, and the O–Sn–Cl moiety was observed in some places. Moreover, maybe some released chloride ions were trapped on the surface or in the lattice of nano-silica.

**FIGURE 2:**
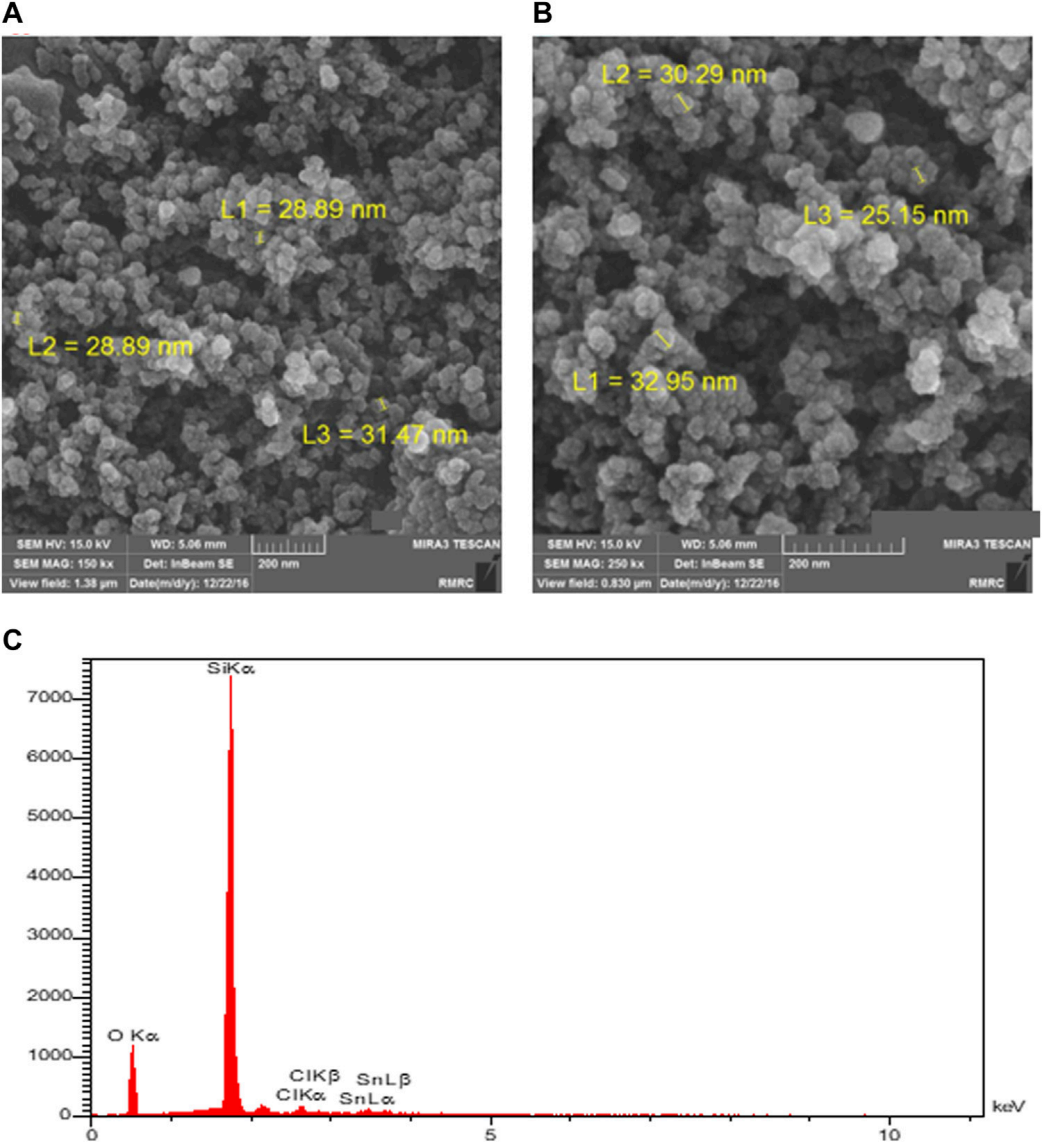
FESEM images of the prepared **(A)** and used **(B)** catalyst (after five runs) and the EDS analysis of the prepared catalyst **(C)**.

The catalyst was first analyzed using the field-emission scanning electron microscopy (FESEM) method. The results for the prepared and used catalyst (after five runs) are shown in Figure 2. The FESEM image of the prepared catalyst (Figure 2A) shows the collections of nearly spherical-shaped particles, which represent the tin-modified nano-silica. There are many separated collections, and each collection consists of many loosely aggregated catalyst particles. The particle size area is closely narrow, and most of the particles have diameters around 30 nm (exactly between 25 and 35 nm). The image of the catalyst was also recorded after five runs of the reaction (after the recyclability experiments) to show the changes in the structure of the catalyst (Figure 2B). The electronic microscope images of the used catalyst are nearly similar to those of the fresh catalyst in all aspects, such as particle size, volume, and distance between collections and the shapes of the catalyst’s nanoparticles. We can, indeed, say that in the reaction conditions, the studied catalysts have such stability that their shapes do not change during the reaction.

During the FESEM analysis, the chemical composition of several top layers of the surface could be evaluated using energy-dispersive X-ray spectroscopy (EDS or EDX) analysis. According to the EDS spectra of the prepared catalyst, as shown in Figure 2C, the atomic percent composition of oxygen, silicon, chlorine, and tin are 58.6%, 38.4%, 1.4%, and 0.7%, respectively (the weight percentages are 43.1%, 50.9%, 2.2%, and 3.8%, respectively). These values confirm the successful surface modification of nano-silica with Sn(II) and Sn-Cl moieties, and approximately one percent of the surface of the silica was decorated with tin(II) ions.

**FIGURE 3:**
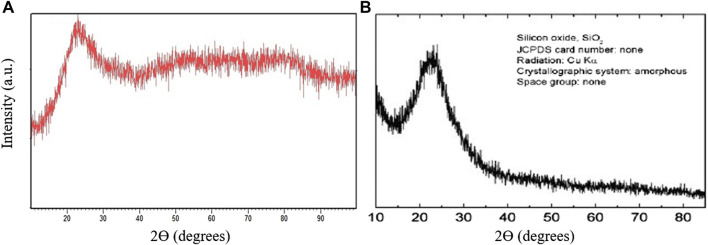
XRD pattern of the prepared catalyst **(A)** compared with nano-silica **(B)**.

The X-ray diffraction (XRD) patterns of the used nano-silica and the prepared catalyst (Sn(II)/nano silica) were recorded, and the results are shown in Figure 3. The XRD pattern of nano-silica shows a characteristic broad peak at *2ϴ* = 23 degrees (in the area between 15 and 35 degrees). This peak is clearly observed in the prepared catalyst, in addition to some broad peaks at 40–90 degrees area. These broad peaks show the presence of amorphous Sn on the surface of nano-silica. Therefore, no obvious crystallinity could be observed for the prepared catalyst.

**FIGURE 4:**
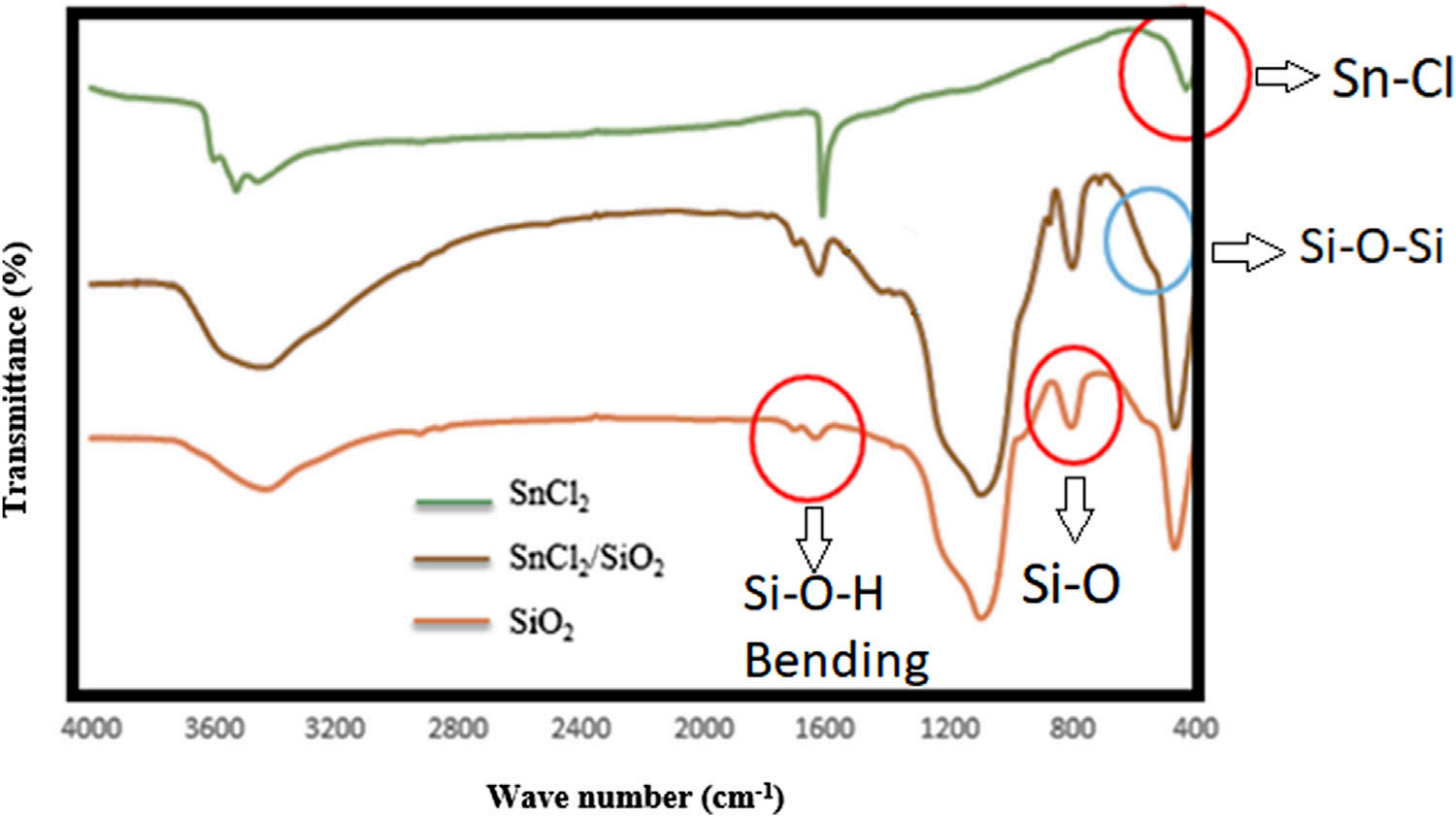
FT-IR spectra of SnCl_2_, nano-silica, and Sn(II)/nano silica (the catalyst).

As the final analysis of the catalyst, its bonding structure was analyzed with Fourier-transformed infrared (FT-IR) spectroscopy, and the results were compared with the FT-IR spectra of the catalyst’s ingredients (SnCl_2_ and nano-silica). A comparative aggregated one-image of these three spectra is shown in Figure 4. The characteristic bands of both silica and SnCl_2_ can be obviously observed in the prepared catalyst. The Sn–Cl stretching vibration at 400–450 cm^−1^, the strong Si–O stretching vibration at 950–1050 cm^−1^, the Si–O–Si bending vibration at 450–500 cm^−1^, and the Sn–O stretching vibration at 600–650 cm^−1^ are the most important absorption bands, which are clearly observed in the presented spectra. The existed band in about 1,620 cm^−1^, which could be observed in all spectra, belongs to the O–H bending, and the broad band after 3,000 cm^−1^ is related to the O–H stretching vibration. This bond exists on the surface of the silica and also in the water molecules of the used tin salt (SnCl_2_.2H_2_O).

The prepared and characterized catalyst was used for the synthesis of dibenzoxanthene derivatives. First, the model reaction between benzaldehyde and β-naphthol was designed to optimize the reaction conditions. The results of 16 optimization experiments, including the reaction conditions and the yield of the product (isolated yield), are listed in Table 1. For optimization of each reaction parameter, a separate part was defined, and the optimized parameters were determined in a bold font. As outlined in the first part of this table (entries 1–4), the reaction was performed in catalyst-free conditions, in addition to the use of SnCl_2_, nano-silica, and Sn(II)/nano silica as catalysts (reaction condition: 5%, reflux in ethanol, and 6 h). The best yield was obtained by using Sn(II)/nano-silica as a catalyst (entry 4% and 40%), while in the other cases, 0%–30% yield was only observed using similar conditions. In the second part of Table 1 (entries 5–7), the amount of the selected catalyst (Sn(II)/nano- silica) for optimization is given. In these experiments, 5, 10, and 20 mol percent of the catalyst were used, and the yields of the products were 45%, 60%, and 60%, respectively. Therefore, 10 mol% of the catalyst (entry 6) was selected as the optimized value because of obtaining the highest yield using the least amount of catalyst. The third part (entries 8–11) contains the yields of the product using no solvent, and THF, water, and ethanol as solvents are listed (reaction condition: 10% catalyst, reflux in ethanol, 6 h). The highest yield was obtained using ethanol (entry 10, which is similar to entry 6) as a solvent. Then, two other temperatures (50 and 25°C, entries 12 and 13, respectively) were used to obtain the best reaction temperature, in which both temperatures gave less yields versus the reflux condition, and entry 11 remained the best reaction condition. Finally, the reaction was carried out at different reaction times (1, 3, and 7 h, entries 14–16, respectively), and 3 h was selected as the optimized value (entry 15).

**TABLE 1:** Optimization of the reaction conditions for the model reaction (1 mmol benzaldehyde and 2 mmol β-naphthol in 10 ml solvent). All yields are reported after the separation, purification, and weighting of the product.

Entry	Catalyst	Catalyst amount (mol%)	Solvent	Temperature (°C)	Time (h)	Yield (%)	Optimization process for
1	—	—	Ethanol	Reflux	6	trace	Catalyst
2	SnCl_2_	5	Ethanol	Reflux	6	30	
3	Nano silica	5	Ethanol	Reflux	6	trace	
4	Sn (II)/nano silica	5	Ethanol	Reflux	6	40	
5	Sn (II)/nano silica	5	Ethanol	Reflux	6	45	Catalyst
6	Sn (II)/nano silica	10	Ethanol	Reflux	6	60	amount
7	Sn (II)/nano silica	20	Ethanol	Reflux	6	60	
8	Sn (II)/nano silica	10	-	120	6	35	Solvent
9	Sn (II)/nano silica	10	THF	Reflux	6	0	
10	Sn (II)/nano silica	10	H_2_O	Reflux	6	10	
11	Sn (II)/nano silica	10	Ethanol	Reflux	6	60	
12	Sn (II)/nano silica	10	Ethanol	50	6	25	Temperature
13	Sn (II)/nano silica	10	Ethanol	25	6	0	
14	Sn (II)/nano silica	10	Ethanol	Reflux	1	30	Time
15	Sn (II)/nano silica	10	Ethanol	Reflux	3	60	
16	Sn (II)/nano silica	10	Ethanol	Reflux	7	60	

**SCHEME 1:**
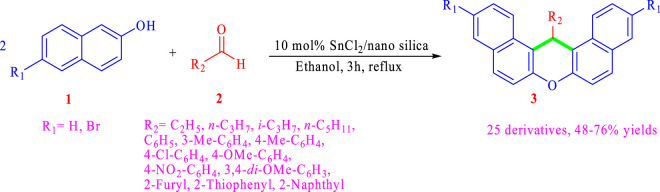
General reaction for the preparation of various *14H*-dibenzoxanthene derivatives.

To examine the generality and versatility of the employed method, the optimized reaction conditions from the previous section, including 10% Sn(II)/nano silica as a catalyst and reflux in ethanol in 3 h, were used in the other reactions to produce various *14H*-dibenzoxanthene derivatives. In these experiments, different aliphatic (propanal, butanal, 2-methylpropanal, and hexanal) and aromatic aldehydes (substituted benzaldehydes, 2-naphtaldehydes, furan-2-carbaldehyde, and thiophene-2- carbaldehyde) were used as aldehyde resources (all of our available aldehydes). Because of the high reaction temperatures, the reactions involving aldehyde with low boiling points (propanal and 2- methylpropanal) were started at smaller temperatures until all the used aldehyde was finished. The reaction with acetaldehyde was not successful because of its very small boiling point. For another reactant, only 2-naphthol and 6-bromo-2-naphthols were available and used for synthesizing *14H*-dibenzoxanthene derivatives. The general reaction for this preparation is shown in Scheme 1.

The complete list of all the products, including melting points, yields, and the corresponding reference number is shown in Table 2. *Via* this method, 25 derivatives of *14H*-dibenzoxathene have been successfully synthesizing using a simple catalyst in high yields (48%–94%). The aromatic aldehydes with electron-donor groups gave smaller yields on average since the aldehyde plays the role of electrophile in the reaction, which will be weakened by electron-donor groups. The yields for the products of aliphatic aldehydes were also small, maybe because of their lower boiling points (that led to evaporating before the completion) or the harder recrystallization process. Moreover, 2-naphthols gave higher yields than 6-bromo-2-naphthols because of the decrease in their nucleophilic potencies due to the presence of the bromine atom. The highest yields were observed in the reaction with 4-nitrobenzaldehyde as an aldehyde resource. Interestingly, the heteroaromatic aldehydes also showed appropriate yields, while, mostly, the reaction involving these aldehydes is complex, with a hard purification process and small yields. In this study, no derivative is obtained from the reactions with 2-substituted benzaldehydes because of the spatial intervention of the substituent, placed at the C2 (ortho) position. In these cases, a complex mixture of the products was obtained, and the purification process was almost impossible. In comparison with the previous related work, in this study, a larger number of derivatives were provided, and the employed catalyst was cheaper and more readily available than most of the previously employed catalysts. Moreover, the preparation of this catalyst was much easier than that found in most of the related reports. A brief comparison between the present study and previous reports was made, and the results are presented in Table 3. According to the data listed in this table, this work is the most comprehensive work (with 25 derivatives), and the products were prepared in the least temperature. Moreover, the employed catalyst is simple, and its value is comparable with that found in the previous works.

**TABLE 2:** Yield, TON, TOF, melting points, and references for the prepared *14H*-dibenzoxanthene derivatives.

	R1	R2	Yield (%)^b^	TON^c^	TOF^d^ (Sec^−1^)	m.p.	Ref.
2a	H	C2H5	53	5.3	2/9 × 10^−2^	128–130	Karimi-Jaberi and Keshavarzi (2010)
2b	H	n-C3H7	62	6.2	3/4 × 10^−2^	151–152	Kumar et al. (2010)
2c	H	i-C3H7	59	5.9	3/3 × 10^−2^	151–152	Kumar et al. (2010)
2d	H	C5H11	64	6.4	3/6 × 10^−2^	97–98	Wang et al. (2014)
2e	H	C6H5	60	6.0	3/3 × 10^−2^	191–192	Kumar et al. (2010)
2f	H	3-Me-C_6_H_4_	55	5.5	3/1 × 10^−2^	204–206	Ghasemnejad-Bosra and Forouzani, (2011)
2g	H	4-Me-C_6_H_4_	59	5.9	3/3 × 10^−2^	234–237	Kumar et al. (2010)
2h	H	4-Cl-C_6_H_4_	65	6.5	3/6 × 10^−2^	291–292	Kumar et al. (2010)
2i	H	4-OMe-C_6_H_4_	57	5.7	3/2 × 10^−2^	212–214	Kumar et al. (2010)
2j	H	4-NO2-C_6_H_4_	76	7.6	4/2 × 10^−2^	328–329	Wang et al. (2014)
2k	H	3,4-di-OMe-C_6_H_4_	48	4.8	2/7 × 10^−2^	195–196	Bhattacharya et al. (2009)
2l	H	2-Furyl	58	5.8	3/2 × 10^−2^	198–200	Shirini and Khaligh, (2012)
2m	H	2-Thiophenyl	61	6.1	3/4 × 10^−2^	179–181	Osyanin et al. (2015)
2n	H	2-Naphtyl	72	7.2	4/0 × 10^−2^	195–198	Shirini and Khaligh, (2012)
2o	Br	C_2_H_5_	50	5.0	2/8 × 10^−2^	134–136	Vaughan and Jha, (2009)
2p	Br	n-C_3_H_7_	55	5.5	3/1 × 10^−2^	152–155	Wang et al. (2014)
2q	Br	i-C_3_H_7_	61	6.1	3/4 × 10^−2^	180–183	Vaughan and Jha, (2009)
2r	Br	C_5_H_11_	58	5.8	3/2 × 10^−2^	oil	Wang et al. (2014)
2s	Br	C_6_H_5_	62	6.2	3/4 × 10^−2^	245–248	Wang et al. (2014)
2t	Br	3-Me-C_6_H_4_	57	5.7	3/2 × 10^−2^	181–183	Pawar et al. (2014)
2u	Br	4-Me-C_6_H_4_	59	5.9	3/3 × 10^−2^	250–251	Wang et al. (2014)
2v	Br	4-Cl-C_6_H_4_	57	5.7	3/2 × 10^−2^	290–292	Wang et al. (2014)
2w	Br	4-OMe-C_6_H_4_	55	5.5	3/1 × 10^−2^	255–260	Pawar et al. (2014)
2x	Br	4-NO2-C_6_H_4_	75	7.5	4/2 × 10^−2^	304–306	Wang et al. (2014)
2y	Br	3,4-di-OMe-C_6_H_4_	51	5.1	2/8 × 10^−2^	184–186	Pawar et al. (2014)

^a^ The reaction conditions for the model reaction (1 mmol benzaldehyde and 2 mmol β-naphthol): 10 mol% catalyst, 10 ml ethanol, reflux, 3 h

^b^ Isolated yield

^c^ Turnover number = the moles of desired product/the moles of catalyst

^d^ Turnover frequency = TON/the reaction time (in seconds)

**TABLE 3:** Brief comparison between the present study and previous reports.

Catalyst	Cat. amount	Time (min)	Temp. (°C)	Solvent	No. of derivatives	Reference
CAN	5 mol%	30	120	Solvent-free	14	Kumar et al. (2010)
Ruthenium/Yb(OTf)3	10 mol%	16 h	110	Toluene	22	Wang et al. (2014)
Sulfated polyborate	10 wt%	∼8	100	Solvent-free	14	Patil et al. (2017)
Boric acid	20 mol%	120	120	Solvent-free	11	Karimi-Jaberi and Keshavarzi, (2010)
Br2-DABCO	17 mol%	∼70	110	Solvent-free	15	Ghasemnejad-Bosra and Forouzani, (2011)
TaCl_5_	10 mol%	60	100	Solvent-free	15	Bhattacharya et al. (2009)
*p*-TSAM.W.	50 mg	5	185	Solvent-free	14	Vaughan and Jha, (2009)
Sn(II)/nano silica	10 mol%	180	76	Ethanol	25	This work

The mechanism of this reaction is well-known and classic. The reaction consists of two consecutive Friedel–Crafts-type reactions between 2-naphthol as an aromatic source and aldehyde as an alkylating agent. This reaction needs a Lewis acid for catalyzing, and silica-supported Sn(II) ion plays this role. It consists of the activation of aldehyde by chelating to the Sn(II) ion and the attack of 2- naphthol from its C1 position to the carbon atom of the activated aldehyde. Then, the proton transfer occurred from the phenolic hydroxy group to the oxygen of aldehyde (which is now converted to the alcohol group). In the second phase, the hydroxy group is activated by chelating to the Sn(II) ion and converted to an appropriate leaving group. Then, the attack of the second 2-naphthol molecule from its C1 position to the carbon atom of the activated alcohol and leaving hydroxy group yields the product after the deprotonation of the phenolic group by the hydroxy group.

**FIGURE 5:**
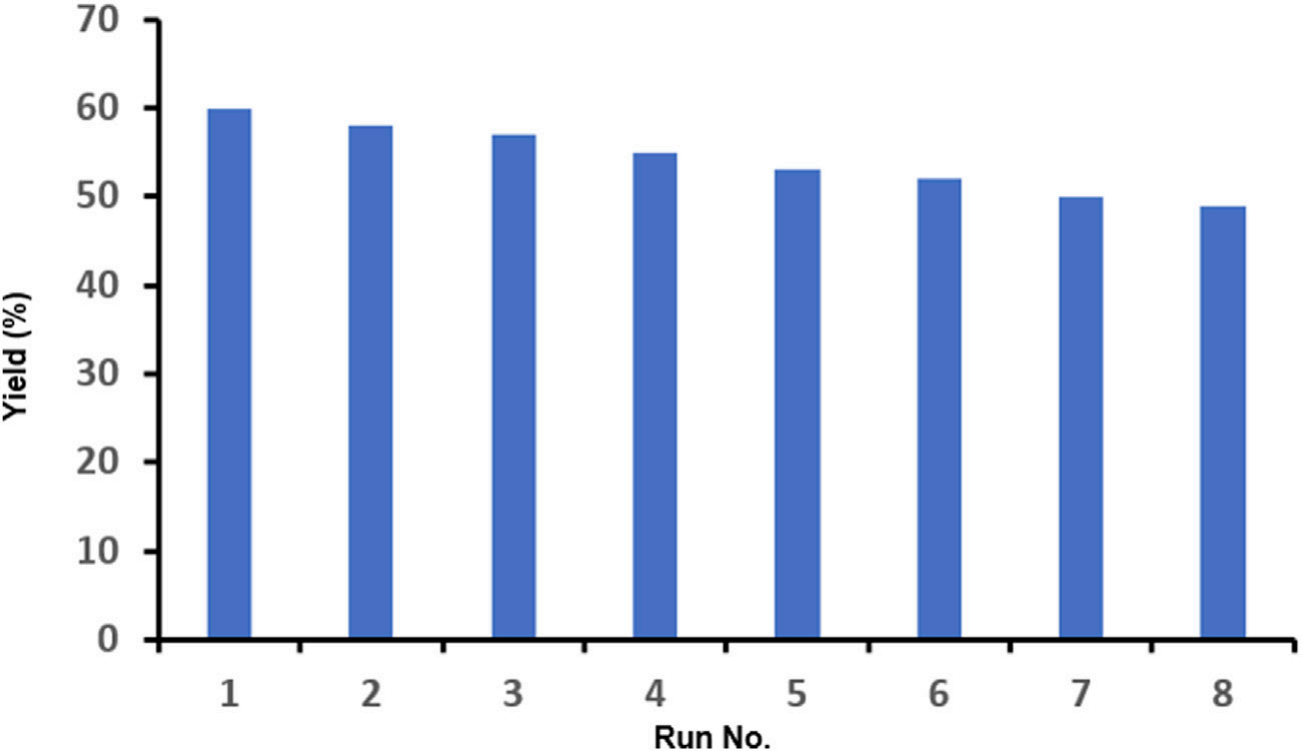
Results of the recyclability experiments for the model reaction at the optimized conditions.

Since one of the essential features of a catalyst is its ability to be recycled and reused in desired reactions, it seems critical to check the presence of this ability in the synthesized nano-catalyst. Therefore, the employed catalyst was reused eight times in the model reaction, and each time, it was hot-filtered, washed with the reaction solvent, dried, and reused. The results of these experiments (Figure 5) indicate that this nano-catalyst maintains its efficiency to a great extent after being reused 8 times, and the yield of the product reached 49% (from 60%) with less than a 20% decrease after eight runs.

## Conclusion

Sn(II)/nano-silica was used as a green and reusable catalyst for the synthesis of 14*H*-dibenzoxanthenes through the one-pot condensation reaction of β-naphthol with various aldehydes. This catalyst is environmentally compatible, low in cost, non-toxic, and easily separable from the reaction medium. The nanocatalyst was characterized by SEM, EDX, FT-IR, and XRD analyses. Twenty-five various xanthene derivatives have been synthesized using ethanol as a solvent at reflux temperature for 3 h with yields between 48% and 94%. This method can be considered one of the most accessible and most appropriate methods for preparing these structures.

## Data Availability

The raw data supporting the conclusions of this article will be made available by the authors, without undue reservation.
